# Venture capital on a shoestring: Bioventures’ pioneering life sciences fund in South Africa

**DOI:** 10.1186/1472-698X-10-S1-S8

**Published:** 2010-12-13

**Authors:** Hassan Masum, Peter A Singer

**Affiliations:** 1 McLaughlin-Rotman Centre for Global Health, University Health Network and University of Toronto, 101 College Street Suite 406, Toronto ON, M5G 1L7, Canada

## Abstract

**Background:**

Since 2000, R&D financing for global health has increased significantly, with innovative proposals for further increases. However, although venture capital (VC) funding has fostered life sciences businesses across the developed world, its application in the developing world and particularly in Africa is relatively new. Is VC feasible in the African context, to foster the development and application of local health innovation?

As the most industrially advanced African nation, South Africa serves as a test case for life sciences venture funding. This paper analyzes Bioventures, the first VC company focused on life sciences investment in sub-Saharan Africa. The case study method was used to analyze the formation, operation, and investment support of Bioventures, and to suggest lessons for future health venture funds in Africa that aim to develop health-oriented innovations.

**Discussion:**

The modest financial success of Bioventures in challenging circumstances has demonstrated a proof of concept that life sciences VC can work in the region. Beyond providing funds, support given to investees included board participation, contacts, and strategic services. Bioventures had to be proactive in finding and supporting good health R&D.

Due to the fund’s small size, overhead and management expenses were tightly constrained. Bioventures was at times unable to make follow-on investments, being forced instead to give up equity to raise additional capital, and to sell health investments earlier than might have been optimal. With the benefit of hindsight, the CFO of Bioventures felt that partnering with a larger fund might benefit similar future funds. Being better linked to market intelligence and other entrepreneurial investors was also seen as an unmet need.

**Summary:**

BioVentures has learned lessons about how the traditional VC model might evolve to tackle health challenges facing Africa, including how to raise funds and educate investors; how to select, value, and support investments; and how to understand the balance between financial and social returns. The experience of the fund suggests that future health funds targeting ailments of the poor might require investors that accept health benefits as part of their overall “return.” Learning from Bioventures may help develop health innovation funding for sub-Saharan African that has combined health, financial, and economic development impacts.

## Background

Since 2000, R&D financing for neglected diseases has increased significantly. In 2008, almost US$3.1 billion was invested in this area, with HIV/AIDS, malaria and tuberculosis initiatives comprising close to three-quarters of investment [1]. There have been innovative proposals for further development of funding mechanisms and sources [2]. However, although venture capital (VC) funding has fostered life sciences businesses across the developed world, its application in the developing world and particularly in Africa is relatively new. Is VC feasible in the African context, to foster the development and application of local health innovation?

VC funds invest in new enterprises, by providing financing and support that helps to scale up promising technologies and business ideas. For decades, venture capital has been used in developed countries to move health technologies from idea to implementation and widespread adoption [3].

There has been discussion of funding for innovative health business models in the African context [4]. While South Africa is a relatively unfamiliar destination for investment from funds based in wealthier countries, it does have solid science, and more capacity and wealth than other countries in sub-Saharan Africa [5]. At the same time, it faces a number of significant health challenges including a high incidence of HIV/AIDS and TB, and its population displays a mix of developed and developing world health profiles.

This paper analyzes Bioventures, the first VC fund focused on life sciences investment in sub-Saharan Africa. Based in South Africa, Bioventures was headed from 2001 onward by CEO Heather Sherwin. This 80 million rand (~$US 12M) fund has invested in 8 home-grown companies since 2002, such as Disa Vascular, a creator of stents for coronary and peripheral artery disease (see Table 1). Despite being a pioneer in the region, Bioventures achieved solid though not stellar returns.

**Table 1:** Bioventures Investment Summary

Investee	Summary	Return
Shimoda Biotech	One drug licensed and on market; 10 in various stages of development and trials; mostly enhanced generics. Company sold to Abraxis Biotech in 2008.	2.5x
Amandla Water Systems	Waste water bioremediation technology worked, but business model failed from long infrastructure tendering cycles and reliance on large water companies.	0x
Disa Vascular	Develops and produces stents for cardiac and other arteries, to keep previously blocked arteries open.	3x, not yet exited.
Synexa Life Sciences	Proprietary bioprocessing technology for production of natural compounds and recombinant proteins.	2.5x, not yet exited.
Electric Genetics	Bioinformatics spin-out from University of the Western Cape. Sector as a whole did poorly.	0x
Mbuyu Biotech	Joint venture with Council for Scientific and Industrial Research, to commercialize the Council's bio-processing technologies.	1x
PlatCo Technologies	Jointly owned with Shimoda Biotech, set up to explore the potential for novel platinum based anti-cancer compounds. Sold to Abraxis in 2008.	7x
Natural Carotenoids SA	Focuses on production and extraction of carotenoids from algae, for food, cosmetics, pharma industries.	1.5x, not yet exited.

We used a case study design. Our analysis is based on semi-structured interviews with key informants, site visits in Cape Town in South Africa, and analysis of peer-reviewed literature, news reports, government and NGO reports, and web sites. We conducted interviews with personnel of Bioventures and related organizations Acorn, Cape Biotech, Disa Vascular, and Real World Diagnostics between October 2007 and June 2009. Bioventures was asked to fact-check the case study; the analysis and interpretation is our own. All quotes are from the interviews unless otherwise noted, and with permission. This study was approved by the Office of Research Ethics of the University of Toronto.

We begin by describing the formation and investment summary of Bioventures. The investment process is then discussed in detail, as this is a key function that venture capitalists must adapt to new settings. One investee, Disa Vascular, is considered in depth. Finally, the challenge of bringing a “health return on investment” into the VC model is discussed, and lessons from the case as a whole considered, including adapting the VC model to work in lower-resource settings to help foster local health innovation.

## Discussion

### Founding and investment summary

Formation of the Bioventures fund was begun in 2000 by Heather Sherwin who had completed her PhD in cell biology in South Africa followed by an MBA. As stated on its web site, "Bioventures believes firmly in the ability of South Africa to develop a quality biotechnology industry that consists of companies that compete with the best in the world [6]." A private placement memorandum to pitch the fund was produced in 2001 and marketed to various investors. Both the International Finance Corporation (IFC) and South Africa’s Industrial Development Corporation invested, attracted by both the developmental and financial potential of domestic biotechnology. According to Sherwin, the fund benefited from approaching investors in the middle of the dot com boom, since biotech was seen as being one of the next big investment areas.

The final trust deed was signed on November 30, 2001. Bioventures was formally established in late 2001, with a relatively small capital base of 80 million rand (approx $US 12 million). As the CEO of the new fund, Sherwin (and the fund itself) was based in Cape Town, South Africa. Paul Miot of Johannesburg was brought in as the CFO; a chartered accountant with an MBA from London Business School, he had worked in banks and was already managing a general private equity fund, and his financial focus was complementary to Sherwin’s scientific background.

Investments started in 2002, and by 2004 the fund had made all its investments. From 2002 to 2009, investments and results were as shown in Table 1. Out of eight investments, final results once all investments were exited (estimated to happen in 2010) were projected to be two outright failures, two break-evens which returned only the initial investment, three which returned between 2 and 3 times, and one which returned between 5 and 7 times. This represents a solid but not spectacular return – a proof of concept that it is possible to do life sciences investments in South Africa and make money.

### Choosing investees

To choose its 8 investments, Bioventures had to go through over 300 proposals; some came from universities, others from local scientists and entrepreneurs. Bioventures benefited from having no local competitors in the sector of life sciences VC. The South African industry itself was small enough that Sherwin knew some of the investees beforehand. The early-stage R&D could require active involvement on the part of the investor, even to the extent of setting up a company to commercialize the science – indeed, Bioventures started three companies to commercialize good technologies.

Bioventures management reported that many proposals were poor quality, and many others too early stage. Although the fund's goal of investing in great science, developing it, and selling the investment for a good price was the same as with developed market funds, Sherwin notes it took more work to develop investees than a developed market fund might typically give: “The perfect business plan with the perfect management team and the perfect science all patented in the right countries just doesn't walk through the door...you've got to be entrepreneurial and create those businesses yourself.”

Since it was difficult for South Africa to compete with India and China in manufacturing, R&D-based businesses were seen by Bioventures management to have more potential, with South Africa’s regulatory environment and perceived quality advantages playing a supporting role. Proactively seeking out investments was necessary. The people involved in the investee companies were often PhDs who left university for the business world, and were sometimes proactively recruited to form companies directly from universities based on the potential of their research.

### Supporting investees

Along with funding, Bioventures supported the companies it invested in with life sciences experience, a network of contacts, and value-added services. Both Sherwin and Miot recalled significant value in simply “being there”: staying in regular contact with investees, learning about human relations and management issues in investees during visits, and using this tacit knowledge to give better, personalized advice and guidance to its investees. As Sherwin put it, “A lot of what I look for when I go visit the businesses – and this is why it is so important in VCs to be on the ground, because you couldn't manage a VC fund remotely – it’s the atmosphere in the business...You see how people are relating to the bosses....If there is a secretary, you know everything in the business from them, and you see the relationships. It's subtle things, it's part of the due diligence that you do on a business...”

A particular challenge for a small fund is spending time on management, investee support, and investment analysis – all activities that may increase overhead costs and thus reduce the fund’s return on investment. This has been identified as one barrier to smaller funds that would invest in global health innovation [7]. For Bioventures, Sherwin had to balance business support and development activities with making a return, yet knew she didn’t have a choice about providing companies with the support they needed – if the support wasn’t provided, the return wouldn’t be made.

As a small fund, overhead had to be kept to a minimum. CFO Paul Miot managed a separate private equity fund and had his own fund management company, which gave him the financial flexibility to become involved part time at Bioventures. As the only full time employee, Sherwin worked on potential deals and investee interaction full time; Miot became more involved when new opportunities came along. Although this lean cost structure was essential to making the fund viable, Miot felt that future funds should be larger, to benefit from economies of scale and get the human resources necessary to fully realize opportunities. Sherwin did note one upside: she thought her investees appreciated the fact that “we live the same lifestyle as our entrepreneurs do”.

### Valuing and exiting investees

A key step when investing into an R&D based business is coming up with a valuation (i.e. estimated worth) of how much the business will be worth in 3, 5, or 10 years. When valuing more mature companies, many valuation methods are used, such as estimating future income streams which are discounted appropriately for risk. But since venture firms like Bioventures invest into new companies, projecting future income is difficult – especially in new sectors as biotechnology was in South Africa.

One method Bioventures used was to agree with an investee company on figures for investment required and future sales. However, two mechanisms were then used to mitigate risk. The investment might be made in parts, with later investments depending on milestones being hit after earlier investments. And if income figures were not hit, then Bioventures would take more shares. (Some caution was required in taking additional shares, in order not to de-incentivize investee management by reducing their ownership stake too far.)

Bioventures aimed to make much of its money back on “exits,” when investments are sold off to purchasers. A key exit strategy was to create IP (Intellectual Property) that would be valuable enough for a US or European company to purchase. These equity exits differ from the debt financing route used to grow a company into a large manufacturing entity; Bioventures perceived this to be a lower-return option in most cases, and one with time scales longer than might be suitable for the fund.

Sherwin reported that an IPO (initial public offering) on local stock exchanges would have been challenging, as local investors tended to be risk averse and less familiar with R&D based health businesses. Another exit strategy was to do an IPO on foreign exchanges, with the Swiss exchange being explored for investee Disa Vascular due to Swiss investors’ long history with healthcare.

Two other investees, PlatCo and Shimoda, were sold to Abraxis Bioscience – a biotechnology company trading on the NASDAQ exchange in the US that has cancer therapeutics as one focus. According to Reuters, Abraxis acquired the full equity of both Shimoda and Platco for an initial payment of US$15 million, plus potential additional payments if specified milestones were met [8].

### Disa Vascular: the investee point of view

Disa Vascular, mentioned in the previous section, was one of Bioventures’ more successful investees – a stent company that built itself from an R&D operation into a full-scale medical device manufacturer.

Gregory Starke, CEO of Disa Vascular and co-founder with Damian Conway, studied orthopedics and cardiology at the University of Cape Town, whose faculty member Christiaan Barnard performed the world’s first heart transplant in the late 1960s. After several years of consulting for local orthopedics companies, he and colleagues decided in late 1999 to develop their own coronary stents, with savings from their consulting work followed by a small angel investment (i.e. small initial investment) in 2000. As Starke recalled, “It was a much bigger leap then we realized…we had a lot of cardiology expertise around that we could get hold of. But on the commercial side, both the production and the ultimate selling of the implant, we were extremely naive and really had no idea of the massive challenges that faced us on actually producing an implant, getting the various certifications etc. and then commercializing it in a professional and normal manner.”

Disa’s management decided early on to pursue European regulatory approval for their coronary stent product, wanting to differentiate themselves in terms of quality perception. Although the device’s South African origins did not in itself pose difficulties for European regulators, Disa Vascular’s management did find it difficult to find local people experienced in taking a new biomedical device through the regulatory process, and later developed this expertise in-house.

Receiving European regulatory approval opened more doors for Disa in international markets, which Starke noted had the unanticipated side effect of causing them to lose focus on the South African market. In practice, they did not have sufficient funds to go to trade shows, do marketing, follow up on leads, and otherwise break into competitive international markets. An experience with an Irish company that licensed the product also turned out poorly, with the Irish company reneging on the agreement once they saw that Disa didn’t have the resources to pursue legal action. Disa’s management rethought their strategy, and came back with new products and investment from Bioventures, and a more mature understanding of markets, branding, and working with distributors.

With a better understanding of “what all the rules were and the ways to get a product to market,” Disa Vascular has had a more typical sales operation for the past several years, with distribution in about a dozen countries in Latin America, Europe, and Asia. As of June 2008, Disa had sold about 6000 stents, with a product line focused on technology for the treatment of coronary and peripheral artery disease, including bare metal stents, catheters, and angiography accessories; the stents had received European certification, and were structurally designed to reduce restenosis (re-narrowing of the artery after stenting due to a build up of scar tissue). A new coated and drug-eluting stent was also undergoing clinical trials [9].

According to Starke, he first met with Heather Sherwin when Bioventures was still in the process of raising funds. In 2002, Disa made an offer to its angel investors that was underwritten by Bioventures, thus enabling the exit of the angel investors while bringing new money into the business. Bioventures invested approximately $2 million, with about $1.3 million coming into the company, and the remainder going to the angel investors. Disa subsequently got another investment of a little less than $US 1 million along with a loan for capital equipment purchase, both from South Africa’s Industrial Development Corporation.

Although these investments let the company survive and grow, and do development and marketing locally, Starke found them to be insufficient to travel to big congresses or take the business global, thus hampering access to new markets. Our analysis revealed other challenges to be getting partners such as cardiologists involved in the testing and development of the stents, doing sales effectively in an organization filled with engineers, coming to terms with ‘soft marketing’ aspects of medical device sales, and a lack of mentorship and local industry experts to network with.

In considering what had drawn Disa to accept funding from Bioventures, Starke reflected that money was naturally the most immediate draw, but that the knowledge, experience, and connections that Bioventures had from being an exclusively life-sciences focused investment firm were also attractive. Conversely, he thought Sherwin saw several attractive features in Disa: clinical credibility, highly motivated management, and a medical device company with shorter investment and regulatory time frames than a drug development company would have. According to Sherwin and Miot, Disa was considered a success story for Bioventures.

Beyond the monetary investment, Bioventures supported Disa in several ways. Both Sherwin and Miot were on Disa’s board of directors. When Disa’s management was looking for additional investment from a European shareholder, they had narrowed options down to Adamant Biomedical (Basel) and Nordic Biotech (Denmark); Sherwin stepped in to look at the term sheets and other details, and helped close the deal with Adamant. Disa paid Bioventures a percentage fee for helping to make the fund investment happen – the only such fee for service asked by Bioventures, according to both Starke and Sherwin.

### Fund performance

The distribution of returns on the investees of the Fund as of 2009 is shown in Table 1. Overall, Sherwin reported that the Fund made money overall, earning a modest but not stellar rate of return from 2001 to 2009 (the exact figure is proprietary).

PlatCo, the investment with the best returns at 7 times initial investment, reportedly made roughly an 80% internal rate of return over the life of the investment. Based on public statements, PlatCo’s buyer Abraxis Bioscence was willing to pay for it because the anti-cancer health technology was seen as having huge potential [8]. Sherwin and Miot felt that in principle Bioventures itself could have taken those products into clinical trials and perhaps made a 20x return instead of 7x, but it didn’t have the time or the money available. PlatCo was almost a virtual company, composed of one researcher, several students, and space rented from a university; as such, its overhead was extremely low.

Bioventures was faced with a landscape in South Africa where there were few established life sciences innovators, be it in biotechnology or in non-generic pharmaceuticals. All of its investees were early stage R&D companies. Challenges included dilution due to inadequate capitalization, investment choices, a fund lifespan which was short for doing biotechnology in a new location, and operating in a challenging environment. The Fund’s short lifespan of only 7 years nominally ended in November 2008, though Sherwin had arranged for a two-year extension period to wind down some investments such as Disa Vascular in a more measured way.

### Balancing financial and social return on investment

Given the overhead and risks involved, Sherwin was ultimately forced to consider whether a typical high “VC rate of return” was achievable, or even a desirable goal to aim for.

Despite Bioventures’ location in South Africa, financial pressures necessitated a focus on developed-world diseases. Diseases whose patients are mostly poor don’t represent attractive financial investment opportunities, absent third-party financial support for innovation. One investee of Bioventures in drug discovery started an HIV program along with a pain treatment program, but was forced to cancel the HIV program for perceived insufficient payback. As Sherwin said, “If you follow your pure VC mandate, you cannot be invested in TB companies, in HIV companies – you're just not going to get your returns.”

Both Sherwin and Miot had thoughts on this balance. Sherwin suggested one analogy for future funds would be microfinance funds, which have pooled investment capital into fund structures, invested the funds into a portfolio of microfinance enterprises, and rewarded the original investors with a combined financial return and social impact return. This “mixed model” investment fund for healthcare companies might invest in those African countries with political stability and populations which can pay for better health care. Such a fund would focus on shorter-term investments that scale up existing health delivery models. For example, investing into a clinic might have a 5 to 7 year time frame to get a loan paid back, as compared to a risky new drug which might require a 10+ year time frame. At least one such fund has been started since Sherwin made this suggestion [10].

Miot thought that wealthy investors and social investment funds might well put money into an “Africa Healthcare Fund”, although the larger potential investors from pension funds might be constrained by real or perceived fiduciary obligations to focus purely on maximizing financial returns. “I think there are a lot of wealthy entrepreneurs and socal investment funds out there who say if you are coming up with a TB [intervention] that is going to help bring down the rate of TB in Africa and we break even on that, that is probably fantastic.” He suggested wealthy investors might like to get involved in social investing, where they made a modest return such as 5%, while being part of something that has made a significant difference in Africa. Pension funds driven by giving returns to their investors might give a small portion of their funds under management, depending on their legal and moral obligations.

### Lessons learned

#### Appreciate scales of time and money

After running Bioventures, Sherwin better appreciated the time and money it takes to see investments through to success: “Timeframe and quantum of money I think has just surprised everyone, and not just in South Africa, but everywhere – just how much money it does take to get it there, to get biotech there.”

Miot felt that access to larger amounts of capital for highly promising investments would be a key feature to be built into future local funds. He suggested partnering with a fund overseas which had more money, so that the local fund would find and grow the investment initially and then exit to the overseas partner fund, which in turn would benefit from a risk-mitigated series of high-quality deals in an emerging market.

Comparing a fund with $10 million to invest against one with $100 million to invest, the overhead as a fraction of funds under management is typically higher for the smaller fund. The smaller fund thus finds it more difficult to provide value-added services, as they may come out of the core bottom line. While investees such as Starke felt that Bioventures had added value, addressing the issue of overhead is essential to making smaller funds work, such as through technical assistance or mentoring provided by third parties on a non-profit basis. Similar issues are likely to arise in the establishment of any large Southern fund focused on investing for health impact.

#### Educate investors about life sciences and emerging economies

Sherwin noted that the IFC (International Finance Corporation), as a large, sophisticated, global investor into Bioventures, had a good understanding of what biotech is and what it requires. This was not the case with some other investors, who compared the performance of Bioventures unfavourably with that of funds in other areas such as IT. Sherwin suggested “I think part of it is us educating them in the beginning, to what they're getting into…in raising [future funds], even if we end up turning down investors or we lose investors, we know that the investors we have really understand what they're doing. It makes a big difference.”

One might question the degree to which Bioventures was truly a “Southern” innovator, given that South Africa is wealthier than its local peers and the modus operandi of Bioventures seems not dissimilar from Western biotech funds. While these are valid points, several features make Bioventures distinct from its Western counterparts. First, the fund size is quite small relative to Western life sciences VC funds. Second, although South Africa has significant wealth, it also contains huge amounts of poverty and need, including one of the most significant HIV/AIDS problems worldwide. Third, the fund’s location in Africa and isolation from both markets and peers form barriers to success. Fourth, local conditions such as market opportunities and external perceptions of South Africa affected the fund’s operational characteristics. Lastly, the CEO faced decisions regarding whether to pursue a typical VC path or to pursue investments oriented more toward local health impacts – the decisions taken by Bioventures, and the corresponding future fund opportunities suggested by Sherwin, form an important learning from the case.

With the above in mind, potential investors from Europe and North America may see risks in investing in emerging economies. However, the solid performance of Bioventures is a positive proof point, and Sherwin believed political and currency risk would be low for offshore investors. Such investors might also be motivated by greater upside potential, given the growing scientific and entrepreneurial strengths of the South. If and when future funds are set up which directly target local health impact, they will be able to benefit from past fund experiences in the region.

#### Learn from peers and previous fund managers

Both of the partners in Bioventures thought that more peer learning and linkages would be useful. Miot speculated, “What would I find useful? I think to know that there is a community out there, to get in contact with similar companies who are maybe further down the path...You are part of the community, and someone could look at presentations that might be public knowledge, etc.” Sherwin similarly felt that networking with domestic and foreign VCs who have solved related challenges a number of times would be helpful. On the investee side, Starke felt there was an opportunity for shared or low-cost health market intelligence, “where we wish to deal with a company in country X but know absolutely nothing about the conditions in that country.”

Sherwin noted the importance of being able to make decisions based on imperfect information, and taking calculated risks. The independence was also a big draw as compared to a corporate finance position, despite a lower salary. Finally, she emphasized the job satisfaction rewards of her work: “I love working with entrepreneurs, I love the fact that I've been at the forefront of this industry in South Africa. I get a real thrill out of seeing companies like Disa go from two guys in a garage to what they are now.” All these factors may be relevant to founders and managers of future health funds.

#### Increase support and follow-on investing for Southern funds

Based on her experience with Bioventures, Sherwin suggested that non-profit funds or services might act as an initial hands-on stage to mentor and grow seed ventures, with high-potential ventures which successfully graduate then receiving follow-on investing. (The Acorn paper in this BMC series describes one such biotechnology incubation service [11].)

Depending on their financial profit potential, this follow-on investing would be either from a mixed-return fund with relatively modest financial return expectations, or from a purely financial fund. This aligns with ideas that have been discussed of designing the character of investment capital to match a combination of financial, health, and development goals [12,13]. More appropriate types of investment could be combined with harnessing educated diaspora populations, as India and China have done so successfully. As Sherwin noted, “There's a large number of Ghanaians and Nigerians and South Africans and Kenyans living abroad who are very highly qualified and skilled. And it's time to start trying to attract these people back.”

## Summary

While Bioventures has been a modest and not stellar success financially, it represents a significant accomplishment: creating a life sciences investment fund in Africa, which has shown proof of concept for investing into R&D based health technologies and creating viable businesses on the ground.

Although South Africa is more economically developed than the rest of sub-Saharan Africa, the fund faced several challenges common to the region, including local capacity constraints and developing IP in the global economy. At the same time, it benefited from positive aspects of the South African context, such as entrepreneurs with personal initiative, private and institutional investment, a positive economic climate, availability of skilled personnel, and a reasonably stable financial and legal climate. In transplanting the venture model elsewhere in Africa, the presence or absence of such factors must be taken into account.

Beyond providing funds, the support given to investees included board participation, contacts, and strategic services. Bioventures had to be proactive in finding and supporting good R&D, and not merely wait for the ideal company to walk through the door. Providing hands-on support to early-stage health ventures posed problems – due to the fund’s relatively small size, overhead and management expenses were tightly constrained. Bioventures sometimes wasn’t able to make follow-on investments, being forced instead to give up equity to raise follow-on investment capital.

With the benefit of hindsight, the CFO of Bioventures Paul Miot felt that partnering with a larger fund might be the way to go. He thought that a small technology-oriented fund such as Bioventures might act as a sort of technology scout and early stage developer, with the larger fund being available for follow-on investments as successful investees grew.

Being better linked to market intelligence and other entrepreneurs was seen as an unmet need – to “people who have done it before.” The experience of the fund also suggests that future health care technology funds targeting ailments of the poor might require investors to accept health benefits as part of their overall “return.” Based on her experience, the CEO of Bioventures has considered several future African fund models balancing health returns with financial returns. (See the Venture Funding article in this BMC series [14].)

While not yet targeting the tougher health challenges facing the continent as a whole, BioVentures has learned lessons about how the traditional VC model might change to tackle those health challenges, yet still retain the risk-accepting spirit that has turned promising ideas into reality in other technological fields. Lessons learned include how to raise funds and find “educated investors”; how to select, value, and support investments; and how to evaluate different models for investment funds targeting health products and services.

In the broader African context, few if any sub-Saharan countries currently have a sufficient scientific base to support a large fund investing solely in early-stage R&D for advanced health technologies. However, there may be scope for funds which invest in a wider range of health technologies, which invest across multiple countries to find sufficient promising technologies, and which provide support, technical assistance, and financing links to help boost domestic African innovation [13]. Similarly, funds which have already been started on the health delivery side suggest the potential viability of co-investing in health technologies along with health delivery systems. The challenge facing those who would invest in health innovation in Africa is to learn from examples like Bioventures, and develop viable health innovation funding that combines health, financial, and economic development impacts.

## Competing interests

None declared.

## Authors' contributions

HM and PAS contributed to the concept and design of this study, participated in site visits, analyzed the findings, and participated in manuscript development.
